# Biological Activities, Health Benefits and Synthesis of Equol and Its Derivatives: A Comprehensive Review

**DOI:** 10.1002/fsn3.71443

**Published:** 2026-01-09

**Authors:** Jiao‐Jiao Zhuo, Bing‐Juan Li, Meng‐Ran Tian

**Affiliations:** ^1^ Tianjin Key Laboratory of Food and Biotechnology, Department of Biotechnology and Food Science Tianjin University of Commerce Tianjin China

**Keywords:** biological activities, equol, glycosylated equol, hydroxylated equol, synthesis

## Abstract

Equol is a metabolite transformed from daidzein by intestinal microorganisms and has received extensive attention due to its various pharmacological activities and therapeutic potential. In recent years, with the in‐depth study of the biological activity of equol, the development of its derivatives has gradually become a research hotspot. These derivatives may exhibit stronger biological activities or more extensive pharmacological effects through structural modification or functional optimization. This review describes the research progress of equol and its derivatives in recent years, with a focus on the expansion of the synthetic mechanism and pathway of equol and its derivatives, and the exploration of the biological activities and functional development of equol and its derivatives. These advances will lay the foundation for the development of equol based nutrition and targeted therapy, thus providing new ideas and methods for the treatment of related diseases.

## Graphical abstract

Biological activities, health benefits and structures of equol and its derivatives.

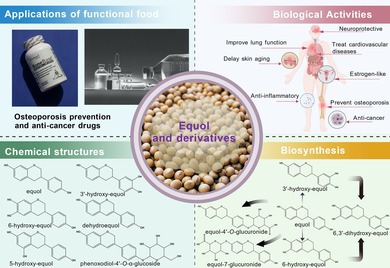

## 1 Introduction

Soybeans, renowned for their abundance of isoflavones, have garnered considerable interest for their nutritional significance and health‐promoting properties. The gut microbiota can metabolize isoflavones found in soybeans and soy products to generate diverse bioactive compounds, with equol emerging as a particularly notable compound due to its unique physiological functions. Recent years have seen an increased focus on elucidating the biosynthetic pathway of equol and understanding its mechanisms of action in various disease models. This has led to a growing recognition of the potential benefits of equol in preventing and treating cardiovascular diseases, osteoporosis, certain cancers, and mitigating menopause symptoms (Choi et al. 2025; Pinaffi‐Langley et al. 2024). The research on derivatives of equol has further broadened the potential applications in this field. Through structural modifications of equol, such as the introduction of diverse functional groups or alterations to the side chain structure, adjustments can be made to its biological activity, pharmacokinetic properties, and receptor affinity. This approach facilitates the development of novel compounds with enhanced drug efficacy or tailored functionalities. This study aims to systematically summarize the research progress of equol and its derivatives, explore their synthesis methods, biological activities, and application prospects in the fields of health food and medicine, and furnish a theoretical foundation for future research and development.

## 2 Introduction to the Structure of Equol and Its Derivatives

Equol, a biphenolic compound, was initially identified and termed by Marrian and Haslewood in 1932 in the urine of pregnant mares (Marrian and Haslewood 1932). Chemically identified as 7‐hydroxy‐3‐(4′‐hydroxyphenyl)‐chroman, equol possesses a molecular formula of C_15_H_14_O_3_ and a molecular weight of 242.27. Equol is characterized as a white to beige powder that is non‐polar and insoluble in water, exhibiting high lipophilicity and a melting point of around 189°C–190°C (Axelson et al. 1982). Under acidic conditions, equol demonstrates instability and susceptibility to degradation. The presence of an asymmetric carbon at the C‐3 position within its molecular framework gives rise to two chiral isomers of equol: *R*‐equol and *S*‐equol. In humans and animals, *S*‐equol predominantly represents the form in which equol exists.

**FIGURE 1:**
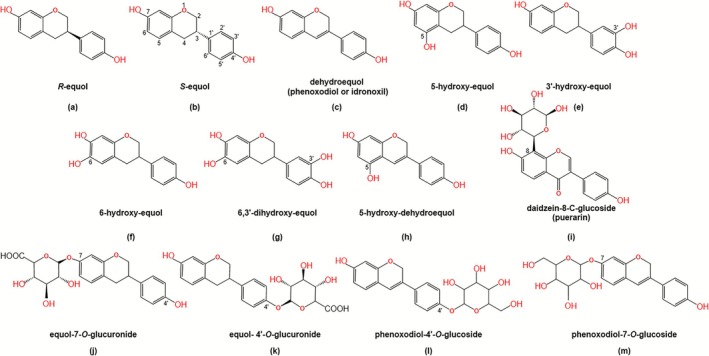
Chemical structures of equol and its derivatives.

Hydroxylated equol compounds encompass a group of substances, including 3′‐hydroxy‐equol, 6‐hydroxy‐equol, 6,3′‐dihydroxy‐equol and 5‐hydroxy‐equol (Figure 1), each synthesized via distinct biosynthetic pathways and possessing antioxidant, estrogenic, and antibacterial activities (Nozawa et al. 2022). Among these, 5‐hydroxy‐equol emerges as a product of genistein transformation by intestinal microbiota, exhibiting heightened antioxidant capacity (Matthies et al. 2009). In contrast, 3′‐hydroxy‐equol and 6‐hydroxy‐equol are synthesized by the monooxygenases. While both compounds demonstrate robust antioxidant efficacy, they exhibit limited estrogenic activity. Notably, 6‐hydroxy‐equol displays significant antibacterial properties against *Escherichia coli* . In addition, physiological activity is closely related to the position and quantity of hydroxyl groups, with an increase in the number of hydroxyl groups notably enhancing antioxidant efficacy beyond that of equol. The synthesis of 6,3′‐dihydroxy‐equol, achieved through the sequential action of the aforementioned monooxygenases, results in a compound with the most potent antioxidant activity, attributed to the presence of two catechol groups in its structure (Nozawa et al. 2022).

Glycosylated flavonoids have attracted much attention due to their significant biological activity, stability, and superior water solubility. The reported equol glycosides such as equol‐4′‐*O*‐glucuronide and equol‐7‐*O*‐glucuronide. These compounds are the phase‐II metabolites of equol in the enterocyte and liver (Giménez‐Bastida et al. 2024; Zhang et al. 2022). The significant changes of equol‐4′‐*O*‐glucuronide in donkeys during pregnancy suggest that it may play an important role in hormone regulation, metabolic support, and the interaction between the gut microbiota and host health (Zhang et al. 2022). This finding could provide valuable insights into the function of equol metabolites during human pregnancy. Equol‐7‐*O*‐glucuronide exhibits anti‐angiogenic activity and may be beneficial to cardiovascular health (Giménez‐Bastida et al. 2024).

In addition, the dehydrooxidation product of equol, dehydroequol (Figure 1), also known as phenoxodiol or idronoxil (4′,7‐dihydroxyisoflav‐3‐ene), possesses a molecular formula of C_15_H_12_O_3_, and a molecular weight of 240.25, exhibiting low water solubility (Constantinou and Husband 2002). This isoflavone is an intriguing regulator of multiple signal transduction pathways. Over the last two decades, a large number of studies have shown that it has the potential to synergize with or complement a wide range of cancer therapies (Porter et al. 2020). However, the low bioavailability of dehydroequol in humans severely limits its development. This is most likely due to its extensive glucuronidation occurring within intestinal cells after being absorbed by the gut. Evidence suggests that even when administered intravenously and bypassing intestinal absorption, the half‐life of its free form in the systemic circulation is extremely short (about 0.67 h), and over 90% of the drug exists in the form of glucuronic acid conjugates (Howes et al. 2011). This indicates that dehydroequol undergoes rapid and extensive metabolism in the body. Therefore, after the dual first‐pass metabolism of dehydroequol in the intestine and liver, the amount of its free protodrug that can enter the systemic circulation will be minimal, thus severely limiting its bioavailability. To overcome this bottleneck, C‐glycosides may represent a key, alternative strategy. Research on puerarin (an isoflavone C‐glycoside) derived from kudzu root has confirmed that it can be absorbed in its intact form through Na^+^‐dependent glucose in combination with SGLT1 transporters and widely distributed to target tissues (Prasain et al. 2009). More importantly, the C‐glycosides were excreted mainly in their original form in bile, and the degree of phase II metabolism was low, which directly proved that the C‐glycoside structure could effectively avoid the first‐pass metabolism. In addition, the development of 5‐hydroxy‐dehydroequol, phenoxodiol‐4′‐O‐glucoside, and phenoxodiol‐7‐O‐glucoside can also effectively improve their bioavailability. This suggests that the development of dehydroequol derivatives is essential to overcome its metabolic bottleneck and achieve efficient drug transformation (Seo et al. 2022).

## 3 Health Benefits of Equol and Its Derivatives

### 3.1 Antioxidant Activity

The antioxidant capacity of equol surpasses that of its precursor daidzein primarily through scavenging free radicals and enhancing antioxidant enzyme activity (Bešlo et al. 2022). Equol effectively mitigates DNA breakage risk and diminishes oxidative stress by virtue of its electron acceptor capability for free radical elimination (Pietta 2000). Furthermore, equol prevents low‐density lipoprotein oxidation by suppressing superoxide radical generation and augmenting free nitric oxide production (Hwang et al. 2003). In macrophages, equol reduces malondialdehyde content, increases reduced glutathione concentrations, and protects cells from lipopolysaccharide (LPS)‐induced oxidative stress (Gou et al. 2015). Equol and dehydroequol are rendered inactive by photoproducts to counteract oxidative damage induced by UV radiation (Widyarini et al. 2001). Preconditioning with equol markedly diminishes oxidative stress markers and boosts antioxidant enzyme function, thereby positively influencing neurological protection and cognitive enhancement (Choi 2009; Sekikawa et al. 2022). Moreover, the antioxidant properties of equol hold promise for contributing to its anti‐aging effects. For example, at a defined concentration, equol demonstrates a substantial enhancement in oocyte embryo developmental competence, mitigating spindle, chromosome, actin impairment, and diminished mitochondrial function, thereby elevating embryo development rates, suggesting a potential attenuation of aging‐related detrimental effects on oocytes (Chen et al. 2025). Hydroxyequol also exhibits antioxidant activity. Specifically, 5‐hydroxy‐equol plays an antioxidant role by inhibiting free radicals generated by metal ions such as Fe (II) and Fe (III) in vivo, thereby counteracting oxidative stress (Arora et al. 1998). Furthermore, the researchers evaluated the antioxidant capacity of 3′‐hydroxy‐equol, 6‐hydroxy‐equol and 6,3′‐dihydroxy‐equol through the 2,2‐diphenyl‐1‐picrylhydrazyl (DPPH) free radical scavenging experiment (Table 1) and observed a marked enhancement in antioxidant efficacy with an increase in the hydroxyl group count, with 6,3′‐dihydroxy‐equol displaying the highest antioxidant activity (Nozawa et al. 2022). This heightened antioxidant capacity is credited to the catechol structure within its molecular framework, facilitating efficient free radical scavenging and cellular protection against oxidative harm, underscoring the close relationship between the number of hydroxyl groups and antioxidant performance.

**TABLE 1:** The antioxidant activity of hydroxyequol was determined by DPPH scavenging of free radicals.

Compound	IC_50_ (μM)	TEAC	EC_50_ (μM)
Trolox	249 ± 19	1	/
Equol	5.20 × 10^4^ ± 1.00 × 10^4^	4.99 × 10^−3^ ± 1.04 × 10^−3^	188
5‐hydroxy‐equol	/	/	9.12
3′‐hydroxy‐equol	250 ± 38	1.02 ± 0.14	7.51
6‐hydroxy‐equol	233 ± 24	1.08 ± 0.10	10.5
6,3′‐dihydroxy‐equol	112 ± 15	2.28 ± 0.33	/

*Note:* IC_50_ refers to half maximal inhibitory concentration. TEAC refers to trolox equivalent antioxidant capacity. EC_50_refers to concentration for 50% of maximal effect. Trolox is a water‐soluble analogue of vitamin E. Among them, the calculation method of TEAC is to divide the IC_50_ value of Trolox standard by the IC_50_ value of each compound. The above data are the results of in vitro experiments.

Abbreviations: EC_50_, concentration for 50% of maximal effect; IC_50_, half maximal inhibitory concentration; TEAC, trolox equivalent antioxidant capacity; Trolox: 6‐Hydroxy‐2,5,7,8‐tetramethylchroman‐2‐carboxylic acid.

### 3.2 Estrogen‐Like Activity

Due to its structural resemblance to estrogen, equol exhibits estrogenic properties and functions akin to estrogen. Its activity surpasses that of its precursor soybean isoflavone, playing a pivotal role in managing estrogen‐dependent conditions. *S*‐equol displays a notable affinity for estrogen receptor‐β (ER‐β), with significantly higher binding affinity than for ER‐α (Muthyala et al. 2004). Conversely, *R*‐equol exhibits diminished binding affinity for ER‐α and ER‐β (Table 2). By modulating estrogen levels in the body, equol effectively mitigates the risk of various diseases, such as improving lung function, reducing the risk of asthma, alleviating menopausal symptoms, and preventing hair loss (Brotzu et al. 2019; Uesugi et al. 2004; Zhang et al. 2024). Clinical trials have validated the favorable outcomes of equol and its precursors, particularly in postmenopausal women with type 2 diabetes, leading to a significant decrease in fasting insulin levels, enhanced insulin sensitivity, and sustained blood glucose stability over the long term (Jayagopal et al. 2002). Similarly to equol, dehydroequol exhibits selectivity for ER‐β and serves as the most potent ERα/β agonist with neuroprotective properties (Cho et al. 2021). The estrogenic attributes of dehydroequol inhibit acute endocardial proliferation in mice, safeguard against arterial wall injury, and evoke vasodilatory effects (Shen et al. 2006). The different hydroxylation positions affect the binding mode with estrogen receptor by changing the molecular hydrophobicity, hydrogen bond interactions, and spatial configuration, thereby determining varied estrogen agonistic or antagonistic activities for each derivative. Notably (Table 2), 3′‐hydroxy‐equol and 6‐hydroxy‐equol have weak estrogen‐like activity, with markedly lower binding affinities to estrogen receptors (ER‐α and ER‐β) compared to *S*‐equol (Song et al. 2022). 5‐hydroxy‐equol displays estrogen‐like activity alongside potent antagonistic properties, but its affinity preference for ER‐α and ER‐β is opposite to that of *S*‐equol (Lee et al. 2017). In recent years, the estrogenic properties of equol and 5‐hydroxy‐equol have attracted extensive attention in the food industry, leading to the successful development of related products (Zhang et al. 2022). Moreover, glycosylated products of equol also show estrogen‐like activity. Equol‐7′‐O‐glucuronide and equol‐4′‐*O*‐glucuronide are found to be the most significantly different in plasma metabolites during pregnancy, playing pivotal roles in bile secretion, ATP‐binding cassette (ABC) transporters, and steroid hormone biosynthesis. These metabolites circulate throughout the body, stimulating hormone production that, in turn, modulates immunity and metabolism essential for overall health.

**TABLE 2:** Analysis of binding affinity and functional activity of different compounds to estrogen receptors ER‐α and ER‐β in vitro.

Compound	*K*_i_ (nM)		*β*/*α*	EC_50_ (nM)		*β*/*α*
	ER‐α	ER‐β		ER‐α	ER‐β	
Estradiol	0.2	0.5	0.4	0.021	0.11	0.19
Daidzein	2000	1300	1.5	250	100	2.5
Genistein	1200	6.7	180	80	6.6	12
S‐equol	200	16	13	85	65	1.3
R‐equol	50	170	0.29	66	330	0.20
5‐hydroxy‐equol	150	340	0.44	/	/	/
3′‐hydroxy‐equol	/	/	/	2.06 × 10^4^	1.79 × 10^4^	1.15
6‐hydroxy‐equol	/	/	/	1.02 × 10^5^	1.02 × 10^5^	1

*Note:* *β*/*α*: The degree of preference of compounds for ER‐β versus ER‐α was compared. *β*/*α* ratio > 1 indicates selectivity for ER‐β. *β*/*α* ratio < 1 indicates selectivity for ER‐α.

Abbreviations: EC_50_, concentration for 50% of maximal effect; *K*i, inhibition constant.

### 3.3 Anticancer Activity

Equol and its derivatives exert anticancer effects through various mechanisms encompassing epigenetic regulation, cell cycle regulation, and cell proliferation inhibition. Equol elicits an upregulation of microRNA‐10a‐5p expression, suppresses the PI3K/Akt signaling pathway, orchestrates the apoptosis cascade, and impedes the cell cycle by regulating the expression of CDK2/4, Cyclin D1/E1 and P21 (Kaufman‐Szymczyk et al. 2025; Tuli et al. 2022). In HeLa cells, the knockout of PAPD5 intensifies equol's efficacy in restraining cancer cell proliferation (Yamashita et al. 2022). Furthermore, derivatives like dehydroequol, optimized structurally through chemical synthesis, exhibit enhanced water solubility and reduced in vivo metabolism, thereby markedly enhancing bioavailability. This optimization enables more efficient targeting of tumor cells, augmenting their therapeutic efficacy. In vitro experiments, dehydroequol showed significant inhibitory effects on a variety of tumor cell lines. For example, the half inhibitory concentration (IC50) of dehydroequol on liver cancer cell Huh‐7 was 3.15 ± 0.03 μM, while its derivative 3‐(2,5‐dimethoxyphenyl)‐2H‐chromen‐7‐ol (A10) exhibited a more potent inhibitory capability with an IC50 of 2.00 ± 0.04 μM (Yang 2023). The mechanism of action of dehydroequol includes inhibiting cell proliferation, inducing cell apoptosis, blocking cell cycle in G2/M phase, inhibiting cell migration and invasion, regulating oxidative stress, and reducing mitochondrial membrane potential. While clinical trials have demonstrated good tolerability, the therapeutic efficacy remains constrained, underscoring the necessity for further investigations into its pharmacokinetics and in vivo performance to optimize clinical utility. Moreover, dehydroequol exerts inhibitory effects on topoisomerase II activity, inducing DNA replication damage in tumor cells and impeding cell division (Constantinou and Husband 2002). It can also inhibit flip expression through the Akt pathway, activate caspase‐8, and promote tumor cell apoptosis (Kamsteeg et al. 2003). Through molecular hybridization technology, the combination of dehydroequol with propranolol significantly augments antiproliferative and antiangiogenic activities (Yee et al. 2013). Furthermore, it enhances the antitumor efficacy of 5‐fluorouracil, oxaliplatin, and gemcitabine while mitigating the risk of gallbladder cancer (Li et al. 2014; Yaylaci et al. 2020). 5‐hydroxy‐equol exerts anticancer effects by suppressing the proliferation, migration, and invasion of liver cancer cells (Gao et al. 2018). Furthermore, isoflavones and their metabolites facilitate the clearance of 17β‐estradiol (E2) by modulating the glucosylation of E2 in liver microsomes, thereby diminishing its carcinogenic risk (Pfeiffer et al. 2005). These findings underscore the multifaceted anticancer mechanisms of equol and its derivatives, offering a novel avenue for cancer treatment research.

### 3.4 Anti‐Inflammatory Activity

Equol and its derivatives exert significant anti‐inflammatory effects through diverse molecular mechanisms, encompassing the modulation of crucial protein levels, suppression of pro‐inflammatory signaling pathways, and amelioration of metabolic dysregulation. In macrophages, equol effectively regulates NLRP3 inflammasome activity, subsequently diminishing nitric oxide (NO) and prostaglandin E2 (PGE2) expression levels, thereby inhibiting LPS‐induced inflammatory response (Márquez‐Flores et al. 2024). In addition, equol demonstrates the capacity to impede interleukin‐6 (IL‐6) and its receptor expression at inflammatory loci, culminating in the reduction of C‐reactive protein (CRP) and tumor necrosis factor‐α (TNF‐α) levels in vivo (Li et al. 2023; Lin et al. 2016). In the field of food and nutrition, bioconverted soy milk is a functional food with great potential. The core lies in leveraging the synergistic effect of tannase and specific probiotics to convert soybean isoflavones into more bioactive aglycones, such as daidzein (Hiramatsu et al. 2020). These aglycones are not only key substances that directly regulate intestinal inflammation but also precursors for the conversion into the highly active metabolite equol. However, this transformation relies on a specific gut microbiota (i.e., “equol producers”), and this ability is relatively lacking in most populations without early soy product intake habits, with less than 20% of Americans being equol producers (Virk‐Baker et al. 2014). Therefore, simply consuming aglycones does not guarantee an increase in equol levels in the body. It is worth noting that studies have identified effective solutions, such as using lactic acid bacteria in fermentation or consuming soy germ formula foods, which can significantly increase plasma equol concentrations in 69% of subjects (including some initial non producers) (Clerici et al. 2007; Ruiz de la Bastida et al. 2021). A large number of studies have confirmed that the resulting equol and its various derivatives have significant anti‐inflammatory potential. Notably, the moderate consumption of isoflavones can elevate equol levels in the body, consequently modulating unsaturated fatty acid content and diminishing the accumulation of proinflammatory metabolites. This alteration in metabolic pathways contributes to the mitigation of inflammation (Shrode et al. 2022). Dehydroequol exerts anti‐neuroinflammatory effects by inhibiting LPS‐activated microglia, reducing neuronal apoptosis, and inhibiting MAPK and JAK/STAT signaling pathways (Yoon et al. 2023). Additionally, glycosylated derivatives of equol showcase anti‐inflammatory potential, exemplified by equol‐4′‐*O*‐glucuronide efficacy in treating gouty arthritis through the inhibition of aberrant xanthine oxidase activity, reduction of serum uric acid levels, and amelioration of energy metabolism disorders (Wang et al. 2019). Meanwhile, equol‐7‐*O*‐glucuronide has been observed to notably stimulate osteoblast activity, exert anti‐inflammatory effects, and enhance bone health (Lee et al. 2017). These findings underscore the multi‐target biological activities of equol and its derivatives in anti‐inflammatory therapy, offering a novel strategic approach and potential application pathway for addressing inflammatory conditions.

### 3.5 Antibacterial Activity

Recent advancements in research have elucidated the antibacterial properties of equol and its derivatives. Investigations indicate that equol exerts inhibitory effects on various pathogenic microorganisms, encompassing both gram‐positive bacteria (such as *Staphylococcus aureus* ) and gram‐negative bacteria (such as *Escherichia coli* ). The antibacterial mechanism of equol likely involves compromising bacterial cell membrane integrity, impeding biofilm formation, and disrupting bacterial metabolic pathways, with notable multi‐target inhibition observed against *Yersinia enterocolitica* . Studies have demonstrated equol's direct bacteriostatic effects through minimum inhibitory concentration assays, along with the downregulation of biofilm‐related gene *hmsT* and motility‐related gene *flhDC*, thereby impeding bacterial attachment, motility, and diffusion (Kim et al. 2022). Notably, 6‐hydroxy‐equol, a significant derivative of equol, has shown stronger antibacterial activity (Nozawa et al. 2022). Experiments showed that 6‐hydroxy‐equol could significantly inhibit the growth of *E. coli* , with subsequent cell survival investigations confirming its bactericidal nature rather than mere bacteriostatic activity. This discovery offers a fresh outlook on comprehending the antibacterial mechanism of equol and its derivatives. Furthermore, these compounds exhibited inhibitory effects on certain drug‐resistant strains, suggesting a promising avenue for the advancement of novel antibacterial agents. Nevertheless, investigations into the antibacterial properties of equol and its derivatives remain at an early stage, necessitating further exploration of their specific mechanisms of action, dose–response relationships, and clinical application potential. Future research endeavors could delve into elucidating the synergistic interactions of equol and its derivatives with other antibacterial agents, as well as assessing their potential in anti‐infection treatment.

**FIGURE 2:**
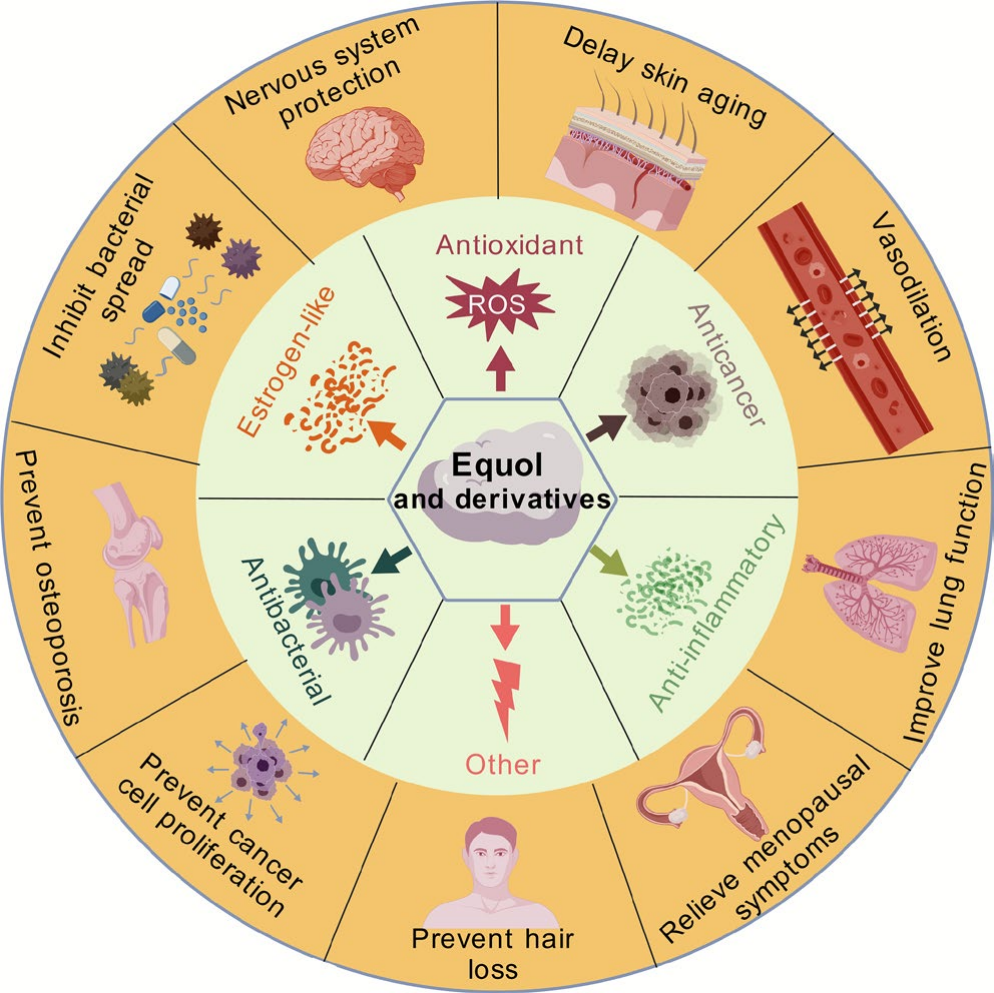
Biological activities of equol and its derivatives.

In summary, equol and its derivatives demonstrate various physiological activities including antioxidant, estrogen‐like, anticancer, and anti‐inflammatory properties (Figure 2). Equol further exhibits a hypoglycemic effect by promoting glucose uptake, AMPK phosphorylation, and GLUT4 translocation in the L6 myotubes (Cheong et al. 2014). *S*‐equol is capable of modulating the TLR4/NF‐κB signaling pathway, thereby mitigating LPS‐induced depressive‐like behavior (Lu et al. 2021). Through coupling with pectin using immobilized laccase, dehydroequol exhibits enhanced stability and reduced toxicity while displaying anti‐angiogenic effects (Yee et al. 2019). Additionally, the glycosylated derivatives of equol also possess anti‐angiogenic properties. For example, equol‐7‐*O*‐glucuronide significantly inhibits the tubular structure formation and cell migration of HAECs at physiological concentrations, blocking VEGF mediated angiogenesis through the inhibition of VEGFR2 phosphorylation and its downstream ERK and Akt signaling pathways (Giménez‐Bastida et al. 2024). Furthermore, dehydroequol has been shown to induce erythrocyte apoptosis and is utilized in the treatment of hemochromatosis (Fink et al. 2019). Serving as a metabolic indicator, 5‐hydroxy‐equol can be employed to assess its precursors' efficacy in inhibiting osteoclast activity and enhancing osteoblast function to prevent osteoporosis (Qiu et al. 2022). These investigations underscore the promising application potential of equol and its derivatives in various disease interventions.

## 4 Biosynthesis of Equol and Its Derivatives

### 4.1 Key Enzymes

Advancements in genetic engineering splicing and sequencing technologies have facilitated biosynthesis as a practical approach for equol production. The biosynthetic pathway from daidzein to equol primarily entails the catalytic actions of four pivotal enzymes: daidzein reductase (DZNR), dihydrodaidzein racemase (DDRC), dihydrodaidzein reductase (DHDR), and tetrahydrodaidzein reductase (THDR) (Soukup et al. 2021). These enzymes play an indispensable role in the biosynthesis of equol.

DZNR plays a pivotal role in the initial reduction step of daidzein, converting daidzein to dihydrodaidzein (DHD). The enzyme belongs to the FMN dependent NADPH oxidoreductase in the old yellow enzyme family (Tsuji et al. 2012). It consists of 644 amino acids, with its N‐terminus featuring a 4Fe‐4S cluster motif (CXXCX_3_CX_12_C), two NADP/NADPH binding motifs (GXGXXG), and an FMN binding domain near the N terminus. The active form of DZNR consists of eight subunits, forming an octameric structure resembling a “spherical four‐leaf clover” with a maximum particle size of 173 Å (Kawada et al. 2018). DZNR can be divided into two types, namely DZNR‐I and DZNR‐II (Hu et al. 2024; Kawada et al. 2018). DZNR‐I is the enzyme represented by Lac 20_92 and DZNR in NATTS, and the consistency of the primary sequence of this class is between 42% and 99%. DZNR‐II is the DZNR represented by K‐07020, and the consistency of the primary sequence of this class is between 70% and 90%. DZNR‐II exhibits both reductase and racemase functions, while DZNR‐I has higher activity for daidzein reductase (Shimada et al. 2010).

DDRC plays an important role in the racemic reaction of *R*‐dihydrodaidzein to *S*‐dihydrodaidzein. The enzyme consists of 158 amino acids with a molecular weight of around 20 kDa. DDRC effectively facilitates the production of *S*‐equol and represents a key enzyme in the stereoselective synthesis of equol (Hu et al. 2024). Studies have demonstrated that the equol conversion rate can reach up to 89.4% in the presence of all four relevant enzymes; however, the absence of DDRC significantly reduces this rate to 15.3% (Deng et al. 2022). This result further confirmed the important role of DDRC in equol biosynthesis. Moreover, experimental data reveal that the presence of DDRC markedly enhances *S*‐equol yield by approximately 5.86‐fold (Shimada et al. 2012).

DHDR belongs to the Short chain Dehydrogenase/Reductase (SDR) family, which relies on the reduced coenzyme NAD(P)H. This SDR enzyme consists of 246 amino acids and contains specific motifs or domains: a cofactor binding motif in the N‐terminal region (TGXXXGXG) (Shimada et al. 2011); domains for stabilizing the central β fold (GXXDXXXNNAG) and SDR active site sequences (Ser and YXXXK) (Oppermann et al. 2003). The catalytic efficiency and substrate preference of DHDR are closely related to pH: under acidic conditions (pH < 7.0), reductase activity prevails, enabling the conversion of *S*‐dihydrodaidzein to (*3S*, *4R*)‐trans tetrahydrodaidzein (THD) in the presence of NADPH (Lee et al. 2016). DHDR is recognized as the rate‐limiting enzyme in the biotransformation process of low‐concentration daidzein (less than 1 mM), with its catalytic efficiency directly impacting the final equol yield. The application of enzyme engineering technology has further enhanced the efficiency of equol synthesis. On the one hand, through the mutation of DHDR, such as the introduction of T169A single point mutation and S118G/T169A double point mutation, the substrate‐enzyme interaction is enhanced, leading to improved substrate binding capability and catalytic efficiency (Qin et al. 2024). Additionally, the P212A mutation in DHDR enhances the selectivity of *S*‐DHD under specific pH conditions, offering theoretical underpinning for the optimization of the equol biosynthetic pathway (Lee et al. 2016).

THDR is a novel dismutase belonging to the reductase family (Kawada et al. 2016). This enzyme does not rely on the coenzyme NAD(P)H and can convert tetrahydrodaidzein (THD) to *S*‐equol without requiring NAD(P)H as a cofactor. THDR exhibits extremely sensitive activity to oxygen and is considered a member of SAM (S‐adenosylmethionine) enzyme (Kim et al. 2010). The catalytic action of THDR is highly stereoselective and can only convert (*3S, 4R*)‐trans tetrahydrodaidzein to *S*‐equol in an irreversible manner. Mutation of THDR, such as P464A, has been shown to notably enhance the production of 5‐hydroxy‐equol, a key derivative of equol, and increase the equol yield in the whole‐cell catalytic system (Lee 2018). This mutation effectively addresses the issue of THDR acting as a limiting enzyme during the conversion of high‐concentration substrates, thereby preventing the accumulation of intermediate products. It serves as a significant catalytic tool for equol biosynthesis under conditions of elevated substrate concentrations.

4‐Hydroxyphenylacetate 3‐hydroxylase (4HPA3H) is a class of two‐component flavin‐dependent monooxygenases from bacteria consisting of an oxygenase component (EC 1.14.14.9) and a reductase component (EC 1.5.1.36). It has a broad substrate spectrum, specifically and catalytically introducing hydroxyl groups to the *o*‐position of phenol analogs to synthesize new phenolic compounds. In the hydroxylation reaction catalyzed by 4HPA3Hs, the oxygenase component is the key part; the oxygenase component is commonly referred to as HpaB (Sun et al. 2024). It has been reported that HpaB_ro‐3_ derived from the *Rhodococcus opacus* enzyme can hydroxylate equol to 3′‐hydroxy‐equol, while the HpaB_pl‐1_ from gram‐negative *Photorhabdus luminescens* and EcHpaB from *Escherichia coli* and the variant T292A enzyme can hydroxylate equol to 6‐hydroxy‐equol (Hashimoto et al. 2019; Song et al. 2022). The simultaneous engagement of HpaB_ro‐3_ and HpaB_pl‐1_ in the reaction enables the efficient synthesis of 6,3′‐dihydroxy‐equol in a single step. These accomplishments not only diversify the array of equol derivatives but also offer novel insights and potential avenues for developing compounds with distinct biological activities. This progress paves the way for extensive research and application prospects in related fields.

Glycosyltransferases (GTs) contain 115 glycosyltransferase families described in the current database (https://www.cazy.org). Uridine diphosphate (UDP)‐glucose glycosyltransferases (UGTs) that usually belong to GT superfamily 1 were reported to glycosylate isoflavonoids possessing free hydroxyl group(s) (Szeja et al. 2017). In recent years, research on equol glycosylation products has received extensive attention. Particularly noteworthy is the utilization of glycosyltransferase MeUGT1 from *Micromonospora echinospora* ATCC 27932 for glycosylating phenoxodiol, a focal point in current investigations (Lee et al. 2022; Seo et al. 2022). MeUGT1, upon heterologous expression in *E. coli* BL21 (DE3), catalyzes the formation of phenoxodiol‐4′‐*O*‐glucoside and phenoxodiol‐7‐*O*‐glucoside at the 4′ and 7 hydroxyl groups of phenoxodiol using UDP‐Glc as a glycosyl donor. The conversion rates are 50% and 30%, respectively. Subsequent investigations demonstrated the diverse glycosyl donor specificity of MeUGT1, showcasing its ability to selectively attach various glycosyl groups to the 4′ hydroxyl group of phenoxodiol. These include glucuronic acid (GlcA), acetylglucosamine (GlcNAc), galactose (Gal), acetylgalactose (GalNAc), and 2‐deoxyglucose (2dGlc). Notably, UDP‐Glc exhibited the highest glycosylation efficiency at 96%, with a production ratio of 60% for 4′‐*O*‐glucoside and 36% for 7‐*O*‐glucoside. These findings unveiled the structural diversity of phenoxodiol glycosylation products, presenting new avenues for potential drug development and broadening its application scope in biomedicine and functional foods.

### 4.2 Biotransformation Strategies

Soy isoflavones (e.g., daidzein, genistein) are metabolized by gut microbes into bioactive compounds like equol and 5‐hydroxy‐equol, but only 20%–35% of western adults produce them in vivo (Langa et al. 2023). This low productivity was particularly evident in a study of postmenopausal women in the United States, which found only 17.5% of equol producers among 143 participants (Virk‐Baker et al. 2014). To overcome the limitations of synthesis in the human body, substantial advancements have been achieved in the investigation of heterologous equol biosynthesis in recent years. By establishing a comprehensive whole‐cell transformation system, including the four key equol synthesis enzymes (DZNR, DDRC, DHDR, and THDR), the production of *S*‐equol could reach 59.0 mg/L/h. The production could be further improved by optimizing enzyme engineering, promoter optimization, and enhancing substrate transport systems (Lee 2018; Lee et al. 2018; Qin et al. 2024; Wang et al. 2024).

Metabolic engineering shows great potential in regulating intracellular redox balance, especially in improving biosynthetic efficiency. In *Escherichia coli*, the optimization of NADPH levels within cells involved redesigning the NADPH reaction pool, upregulating key genes, and disrupting genes influencing NADPH supply. This strategy has achieved remarkable results in a large‐scale experiment in a 5 L fermentor, with *S*‐equol reaching a yield of 3418.5 mg/L and a conversion rate of approximately 85.9% (Deng et al. 2022). Moreover, genetic modification of the *E. coli* Nissle 1917 (EcN) strain, where the SdiA gene was replaced with the K12 strain's SdiA gene, led to a substantial elevation in the intracellular NADPH/NADP+ ratio, further optimizing the redox balance (Wang et al. 2024). The modified strain achieved a substrate conversion rate of 84.8%, resulting in an increased *S*‐equol concentration of 113.7 μM and a 47% yield enhancement compared to pre‐modification levels. These findings conclusively demonstrate that optimizing intracellular redox balance represents an effective strategy for enhancing equol synthesis efficiency, offering valuable insights for related biosynthesis research endeavors.

Due to a growing demand for functional foods, lactic acid bacteria and bifidobacteria have gained importance in the food industry due to their probiotic characteristics and various bioactive compounds (Clavel et al. 2014; Heng et al. 2019; Kim et al. 2010; Kwon et al. 2018). Up to today, approximately twenty lactic acid bacterial strains have been identified and described; they include the genera *Lactobacillus* sp., *Bifidobacterium* sp., *Enterococcus* sp., and *Lactococcus* sp. and could be used for the bioconversion of daidzein into equol. Among them, lactic acid bacteria's potential is being increasingly harnessed because these types of bacteria are often cataloged as generally recognized as safe microorganisms (GRAS) (Kim et al. 2010). Through genetic engineering‐mediated introduction of key enzymes DHDR and THDR, the strain exhibited efficient transformation capabilities, culminating in the synthesis of high concentrations of equol, with levels reaching 241.34 ± 34.56 μM. This synthetic proficiency not only underscores the lactic acid bacteria's efficacy in biocatalysis but also lays a theoretical foundation for industrial production. Furthermore, the versatility of lactic acid bacteria extends beyond this feat. Research indicates that even in the absence of THDR, DHDR can independently facilitate the direct conversion of dihydrodaidzein to dehydroequol. This discovery provides a novel approach for optimizing the synthesis pathway and further expands the application scope of lactic acid bacteria in biosynthesis. In the synthesis of 5‐hydroxy‐equol, the high glycosidase activity of *Bifidobacterium pseudocatenulatum* INIA P815 was effectively leveraged to convert genistein glycosides into free aglycones, leading to the successful synthesis of 5‐hydroxy‐equol under the cooperative action of four enzymes DZNR, DHDR, THDR, and *ifc*A (Langa et al. 2022). This process not only showed the high efficiency of microbial synthesis but also significantly increased the concentration of 5‐hydroxy‐equol (125.54 ± 7.90 μM) through the synergistic effects of DHDR and THDR (Langa et al. 2025). Furthermore, the direct conversion of DHG to 5‐hydroxy‐dehydroequol at concentrations up to 292.34 ± 14.67 μM was achieved by introducing DDRC and DHDR.

Overall, biotransformation remains the dominant approach for the future development of equol and its derivatives. At present, only a handful of enzymes capable of hydroxylating or glycosylating the equol scaffold have been reported. Moving forward, equol derivatives can be generated by engineering existing enzymes, while the discovery of novel biocatalysts capable of introducing regio‐ and stereoselective modifications to equol represents a key research direction.

## 5 Chemical Synthesis of Equol and Related Derivatives

To date, equol synthesis methods exhibit significant variations in precursor selection, reaction pathways, and chemical transformations, broadly divided into five categories. The traditional chemical synthesis method takes phenols or salicylaldehydes as precursors, combined with condensation, reduction, demethoxy and Grubbs‐catalyzed cyclization, yielding between 22.3% and 32.62% (Gharpure et al. 2008; Gupta and Ray 2008; Li et al. 2009). The halogenation and cleavage strategy synthesizes *S‐*equol through chlorine‐bromine substitution, dehydration, and etherification processes, albeit with a relatively low yield of 9.69% (Heemstra et al. 2006). Utilizing the Witting reaction and multi‐step reduction method, *S*‐equol is derived through a sequence involving the Witting reaction for olefin intermediate generation, followed by bromination, substitution, and reduction, resulting in a 23.34% yield (Takashima et al. 2010). The natural product derivatization method employs daidzein as the starting material for *S*‐equol synthesis, involving steps such as hydroxyl protection, hydrogenation, dehydration and chiral catalysis. Among these methods, the Steffan method stands out with the highest yield, reaching 44.46% (Steffan 2010). The amino acid derivatization strategy uses 2,4‐dimethoxybenzaldehyde as the starting material, uses amino acid‐derived chiral auxiliaries to mediate carbonyl compounds, ultimately yielding *S*‐ and *R*‐equol with remarkable efficiency up to 90%, which is the best synthesis method at present (Uemura et al. 2021).

There are three primary synthetic routes of dehydroequol. One route utilizes daidzein as the starting material, subjected to high‐pressure catalytic hydrogenation and dehydration with a palladium/alkaline carrier catalyst to yield the target product (Heaton and Jeoffreys 2005). The method is straightforward but necessitates high pressure. Another approach involves daidzein as the raw material, undergoing a multi‐step reaction including phenolic hydroxyl protection, palladium carbon reduction, alcohol elimination and alkaline deprotection (Zhang et al. 2013). While the steps are relatively simple, the raw material cost is high. The third route utilizes deoxybenzoin as the initial compound, leading to the target product through a multi‐step reaction (Jain and Mehta 1986). This route is cost‐effective but involves relatively more reaction steps.

Although the synthesis methods of equol and dehydroequol are becoming more diverse, challenges persist, including high raw material costs, complex processes, and chiral control difficulties. Future research directions may focus on enhancing yield and optimizing chiral catalyst or enzyme catalytic strategies to boost synthesis efficiency. Efforts could be directed towards advancing green synthesis by minimizing the use of organic solvents and heavy metal catalysts, exploring biocatalysis or microbial synthesis pathways for eco‐friendly and effective synthesis. Additionally, refining chiral selectivity control and enhancing *S*‐equol selectivity can align with pharmaceutical industry demands. Through the deep integration of chemical synthesis and biocatalysis, a breakthrough in efficient, green, and sustainable industrial equol production is anticipated.

## 6 Future Perspectives

With the extensive exploration of the biological properties of equol and its derivatives, mounting evidence underscores their promising applications in estrogen‐like effects, anticancer activities, anti‐inflammatory properties, and neuroprotective functions. Nonetheless, current research faces challenges in optimizing synthesis methods, expanding the structural activity and synthesis pathways of derivatives, and conducting detailed mechanistic analyses. Future research should prioritize the following aspects to advance the utilization of equol and its derivatives in biomedicine and functional food sectors.

Firstly, improving the efficiency and sustainability of synthesis methods is a key focus for future research. Presently, equol synthesis methods encompass chemical and biosynthetic routes. While chemical synthesis approaches are established, they often involve complex multi‐step reactions with low yields, potentially leading to environmental impact through the use of organic solvents and catalysts. Therefore, efficient and environment‐friendly synthesis strategies should be explored, such as introducing chiral catalysts with higher catalytic efficiency, optimizing key reaction steps, reducing the generation of by‐products, and boosting target product yields. Furthermore, enzyme catalysis, as an environmentally friendly synthesis avenue, offers benefits such as high regional and stereoselectivity alongside mild reaction conditions. In recent years, the development of synthetic biology has provided innovative ideas for efficient equol production. These methods involve *Escherichia coli* modification through genetic engineering to optimize the biosynthetic pathway, enhance yield, improve stereoselectivity, and establish sustainable biological manufacturing processes. Secondly, expanding the structure and function of equol derivatives is an important strategy to enhance their pharmacological activity. Previous studies have highlighted the substantial impact of structural modifications on equol's biological effects; for instance, glycosylation can enhance water solubility, while alkylation or acylation can improve cell membrane permeability and stability. Moving forward, the integration of synthetic biology and artificial intelligence offers opportunities to facilitate drug design, predict optimal modification sites, and synthesize derivatives with enhanced activity and selectivity. Simultaneously, enhancing the bioavailability of equol can improve its stability, targeting in vivo, and therapeutic efficacy. In addition, comprehensive elucidation of the equol mechanism is pivotal for future research. Present investigations into equol's anticancer effects primarily center on apoptosis, cell cycle arrest, and antioxidant stress, yet its interactions with intestinal microbes, the immune system, epigenetic regulation, and other facets remain unclear. In the future, the integration of single‐cell sequencing, metabolomics, and artificial intelligence analysis can systematically delineate the in vivo equol pathway, offering a foundation for precision medicine. Subsequent research on equol and its derivatives will advance towards efficient and sustainable synthesis, derivative optimization, and mechanistic analysis. The ongoing advancements in synthetic biology and artificial intelligence are anticipated to drive breakthroughs in industrial production and clinical translational applications of equol and its derivatives, potentially revolutionizing disease prevention and treatment strategies.

## Author Contributions

**Jiao‐Jiao Zhuo:** conceptualization, formal analysis, writing – original draft, writing – review and editing. **Bing‐Juan Li:** conceptualization, project administration, funding acquisition, writing – review and editing. **Meng‐Ran Tian:** conceptualization, data curation, supervision, writing – review and editing.

## Funding

This work was supported by National Natural Science Foundation of China, 31801471.

## Conflicts of Interest

The authors declare no conflicts of interest.

## Data Availability

The data that support the findings of this study are available from the corresponding author upon reasonable request.
